
JOURNAL INFORMATION
==============================
NLM Title Abbreviation: ScientificWorldJournal
Journal ID: ScientificWorldJournal
NLM Journal ID: 101131163
Journal ID: TSWJ

The Scientific World Journal

ISSN: 2356-6140
EISSN: 1537-744X
Publisher: Wiley

ARTICLE INFORMATION
==============================
PMCID: PMC5956397
PMID: 15349540
DOI: 10.1100/tsw.2004.60
Article ID: 538403
Article version: 1
Subjects: Research Article

Testosterone Replacement Therapy

Hellstrom Wayne J.G.
Tulane University Medical Center, New Orleans, LA, USA
Academic Editor: Anthony Atala

Publication date: 2004
Electronic publication date: 2004 Jun 7
Volume: 4
First page: 142
Last page: 149
Copyright: Copyright © 2004 Wayne J.G. Hellstrom.
Copyright year: 2004
License: This is an open access article distributed under the Creative Commons Attribution License, which permits unrestricted use, distribution, and reproduction in any medium, provided the original work is properly cited.
License URL: https://creativecommons.org/licenses/by/3.0/

==============================
Article
*TheScientificWorldJOURNAL* (2004) 4 (S1), 142–149
ISSN 1537-744X; DOI 10.1100/tsw.2004.60

### Testosterone Replacement Therapy

Wayne J.G. Hellstrom, M.D.
*Tulane University Medical Center, New Orleans, LA*

**E-mail**: whellst@tulane.edu

*Previously published in the Digital Urology Journal*

**DOMAIN**: urology

### INTRODUCTION

Testosterone is responsible for normal growth and development of male sex organs and maintenance of secondary sex characteristics. It is the primary androgenic hormone, and its production and secretion are the end product of a series of hormonal interactions. Gonadotropin-releasing hormone (GnRH) is secreted by the hypothalamus and controls the pulsatile secretion of luteinizing hormone (LH) and follicle-stimulating hormone (FSH) by the anterior pituitary. Luteinizing hormone regulates the production and secretion of testosterone by the Leydig cells of the testes, and FSH stimulates spermatogenesis.

When the testes fail to produce normal levels of testosterone, testosterone deficiency results. Hypergonadotropic hypogonadism is caused by primary testicular failure. Testosterone levels are low and pituitary gonadotropins are elevated. In secondary, or hypogonadotropic hypogonadism, there is inadequate secretion of pituitary gonadotropins. In addition to a low testosterone level, LH and FSH levels are low or low-normal.[1, 2] While pre-pubertal hypogonadism is generally characterized by infantile genitalia and lack of virilization, the development of hypogonadism after puberty frequently results in complaints such as diminished libido, erectile dysfunction, infertility, gynecomastia, impaired masculinization, changes in body composition, reductions in body and facial hair, and osteoporosis.[1] In addition to these complaints, mood inventory scores indicate that hypogonadal men report levels of anger, confusion, depression, and fatigue that are significantly higher than those reported by men with normal testosterone levels.[3]

Men with primary hypogonadism (congenital or acquired) or hypogonadotropic hypogonadism are candidates for testosterone replacement therapy, and there are now a variety of products available to treat these disorders. Successful management of testosterone replacement therapy requires appropriate evaluation and an understanding of the benefits and risks of treatment.

### DIAGNOSIS OF TESTOSTERONE DEFICIENCY

Given the variety of causes of testosterone deficiency, a medical and medication history, physical exam, and directed laboratory evaluation are imperative. The medical history should include questions regarding developmental abnormalities at birth, the rate and extent of virilization at the time of puberty, and the current status of sexual function and secondary sexual characteristics, such as beard growth, muscular strength, and energy level. Hypogonadal men have statistically significant reductions in the incidence of nocturnal erections, the degree of penile rigidity during erection, and the frequency of sexual thoughts, feelings of desire, and sexual fantasies.[3] Alterations in body composition, including increases in percent body fat, changes in adipose tissue distribution, and reduction in muscle mass, are frequently seen in hypogonadal men.[4,5] Spinal trabecular bone density is also decreased in men with hypogonadotropic hypogonadism,[6] and hip fractures are more common in hypogonadal men than in normal men.[7]

Initially, hormonal screening is limited to measurement of total serum testosterone, which is obtained in the morning. When the total testosterone level is low and/or the patient complains of reduced libido, a serum prolactin level should also be measured. A high serum prolactin level may indicate pituitary dysfunction and may require consultation with an endocrinologist. Serum LH levels are measured when serum prolactin levels are normal or low to help differentiate intrinsic testicular failure from a pituitary or hypothalamic abnormality. LH is usually high in patients with primary testicular disease. When the serum testosterone level is low and LH is elevated, testosterone replacement therapy is warranted.

### TESTOSTERONE REPLACEMENT THERAPY

Testosterone replacement should in theory approximate the natural, endogenous production of the hormone. The average male produces 4-7 mg of testosterone per day in a circadian pattern, with maximal plasma levels attained in early morning and minimal levels in the evening.[8] However, the subtleties of pulsatile and diurnal rhythms are potentially difficult to imitate, and evidence suggests that different dose response curves exist for different androgen-dependent functions.[9] The clinical rationale for treatment of testosterone deficiency may include:

- stabilizing or increasing bone density
- enhancing body composition by increasing muscle strength and reducing adipose
- improving energy and mood
- maintaining or restoring secondary sexual characteristics, libido and erectile function

### TYPES OF TESTOSTERONE REPLACEMENT THERAPY

Ideal testosterone replacement therapy produces and maintains physiologic serum concentrations of the hormone and its active metabolites without significant side effects or safety concerns. Several different types of testosterone replacement are currently marketed, including tablets, injectables, and transdermal systems.

### Oral Agents

Although elevations in liver function tests and abnormalities at liver scan and biopsy are relatively common in patients receiving oral testosterone,[10] these preparations still constitute roughly a third of the testosterone prescriptions filled in the United States. Both modified and unmodified oral testosterone preparations are available. Unmodified testosterone is rapidly absorbed by the liver, making satisfactory serum concentrations difficult to achieve. Modified 17-alpha alkyltestosterones, such as methyltestosterone or fluoxymesterone, also require relatively large doses that must be taken several times a day.

### Intramuscular Injection

Testosterone cypionate and enanthate are frequently used parenteral preparations that provide a safe means of hormone replacement in hypogonadal men. Testosterone is esterified to inhibit degradation and to make it soluble in oil-based injection vehicles that retain the drug in muscle tissue. In men 20-50 years of age, an intramuscular injection of 200 to 300 mg testosterone enanthate is generally sufficient to produce serum testosterone levels that are supranormal initially and fall into the normal ranges over the next 14 days. Fluctuations in testosterone levels may yield variations in libido, sexual function, energy, and mood. Some patients may be inconvenienced by the need for frequent testosterone injections.[11] Increasing the dose to 300 to 400 mg may allow for maintenance of eugonadal levels of serum testosterone for up to three weeks, but higher doses will not lengthen the eugonadal period.[12]

### Transdermal Systems

Currently, three testosterone transdermal systems are marketed: a system applied to the scrotum that has no permeation enhancers [Testoderm, 6 mg, ALZA Corporation, Palo Alto, CA] and two systems that contain permeation enhancers for application to appendage or torso skin [Androderm 2.5 mg and 5 mg, SmithKline Beecham Pharmaceuticals, Philadelphia, PA; Testoderm TTS, 5 mg, ALZA Corporation, Palo Alto, CA]. Scrotal patches produce high levels of circulating dihydrotestosterone (DHT) due to the high 5-alpha-reductase enzyme activity of scrotal skin.

Clinical studies of transdermal systems demonstrate their efficacy in providing adequate testosterone replacement therapy.[13-15] Skin irritation may be associated with the use of transdermal systems; however, Testoderm and Testoderm TTS caused significantly less topical skin irritation than Androderm in two separate clinical studies.[16,17]

### MONITORING PATIENTS ON TESTOSTERONE REPLACEMENT

Patients on testosterone replacement therapy should be monitored to ensure that testosterone levels are within normal levels. The physician prescribing testosterone replacement should evaluate any changes in the clinical symptoms and signs of testosterone deficiency and should assess for other concerns, such as acne and increase in breast size and tenderness. Serum testosterone levels should be checked three to 12 hours after application of a transdermal delivery system. For patients on injectable testosterone, nadir testosterone levels should normally be obtained at three to four months prior to the next injection. Levels that exceed 500 ng/dL or are less than 200 ng/dL require adjustment of the dose or frequency.

A digital rectal examination (DRE) should be performed and prostate specific antigen (PSA) checked in all men before initiating treatment. These should be repeated at approximately three to six months, and then annually in men >40 years of age. An abnormal DRE, a confirmed increase in PSA >2 ng/mL, or a total PSA >4.0 ng/mL requires urologic evaluation that usually consists of transrectal ultrasonography and sextant prostate biopsies. The hematocrit level should also be checked at baseline, at three to six months, and then annually. A hematocrit >55% warrants evaluation for hypoxia and sleep apnea and/or a reduction in the dose of testosterone therapy. Measurement of bone mineral density of the lumbar spine and/or the femoral necks at one year may be considered in hypogonadal men with osteopenia.

### BENEFITS OF TESTOSTERONE REPLACEMENT THERAPY

A number of benefits of testosterone replacement therapy have been demonstrated, including effects on mood, energy levels, and libido. Long-term follow-up of testosterone replacement in hypogonadal males and a control group indicates that self-assessment of libido was significantly higher ($p<0.0001$) in the testosterone-treated group.[18] Testosterone replacement has also been shown to enhance libido and the frequency of sexual acts and sleep-related erections.[19] Transdermal testosterone replacement therapy, in particular, has been linked to positive effects on fatigue, mood, and sexual function, as well as significant increases in sexual activity.[20] More specifically, testosterone replacement therapy has been shown to improve positive mood parameters, such as feelings of wellness and friendliness, while reducing negative mood parameters, such as anger, nervousness, and irritability.[21] Testosterone replacement is an effective treatment for some depressive symptoms in hypogonadal men and may effectively augment treatment in selective serotonin reuptake inhibitor (SSRI)-refractory major depression.[22] Relative to eugonadal men, hypogonadal men in one study were impaired in their verbal fluency and showed improvement in verbal fluency following testosterone replacement therapy.[23]

Testosterone replacement therapy is also associated with potentially positive changes in body composition. In hypogonadal men, testosterone replacement therapy has demonstrated a number of effects, including an increase in lean body mass and decrease in body fat,[24] an increase in weight,[25] and increases in muscle size.[26] Parenteral testosterone replacement in hypogonadal men resulted in improved strength and increased hemoglobin compared to controls.[27] In another study by Urban and colleagues,[28] testosterone administration also increased skeletal muscle protein synthesis and strength in elderly men. Testosterone replacement with transdermal testosterone delivery systems in HIV-infected men with low testosterone levels has been associated with statistically significant gains in lean body mass ($p=0.02$), increased red cell counts, and improvements in emotional distress.[29] Transdermal testosterone has also been administered to HIV-positive women, yielding positive trends in weight gain and quality of life.[30]

Improvements in bone density have also been shown with testosterone replacement therapy. Increases in spinal bone density have been realized in hypogonadal men,[31] with most treated men maintaining bone density above the fracture threshold.[32] Testosterone replacement in hypogonadal men improves both trabecular and cortical bone mineral density of the spine, independent of age and type of hypogonadism.[33] In addition, a significant increase in paraspinal muscle area has been observed, emphasizing the clinical benefit of adequate replacement therapy for the physical fitness of hypogonadal men. [33]

### CONTRAINDICATIONS TO TESTOSTERONE REPLACEMENT THERAPY

Testosterone replacement is contraindicated in men with carcinoma of the breast or known or suspected carcinoma of the prostate, as it may cause rapid growth of these tumors. Hormone therapy is also inappropriate in men with severe benign prostatic hypertrophy (BPH)-related bladder outlet obstruction. Use of testosterone to improve athletic performance or correct short stature is potentially dangerous and inappropriate. In addition, the hormone does not correct ambiguous genitalia resulting from androgen deficiency during fetal development[34,35] and should not be administered at dosages high enough to inhibit spermatogenesis.

### SAFETY ISSUES WITH TESTOSTERONE REPLACEMENT

Although testosterone replacement may be indicated in the aging male with documented hypogonadism, this hormone should not be administered with the intent of reversing the aging process in men with normal testosterone levels. Testosterone replacement therapy may be associated with azoospermia, lipid abnormalities, polycythemia, sleep apnea, and the potential for prostate changes.

### Azoospermia

The administration of exogenous testosterone as a means of male contraception is under study.[36] In these men, azoospermia usually results within approximately 10 weeks of beginning therapy. Rebound of the sperm count to baseline levels occurs within six to 18 months of cessation, and subsequent fertility has been demonstrated.[37]

### Lipid Abnormalities

Physiologic testosterone replacement is known to reduce total cholesterol, low density lipoprotein (LDL), and high density lipoprotein (HDL) levels,[24] but the extent to which these parameters are affected by treatment varies considerably between studies. While reductions in HDL did not reach significance in a study by Tenover[24] and testosterone replacement was not associated with unfavorable changes in lipid profiles in a separate study by Tan and colleagues,[38] research by Anderson and colleagues[39] suggests testosterone replacement therapy may result in significant reductions in HDL and elevations in blood viscosity. Some authorities recommend that lipid values be followed closely in men receiving testosterone replacement therapy.

### Polycythemia and Sleep Apnea

Polycythemia has been associated with testosterone replacement therapy[24] and is correlated with elevated bioavailable testosterone and estradiol levels.[40] Physiologic replacement with transdermal testosterone, however, resulted in fewer cases of polycythemia than replacement with testosterone enanthate injections.[40] Although the mechanism is unclear, testosterone replacement therapy may also cause or worsen obstructive sleep apnea.

### Prostate Changes

Although PSA is not specific for prostate cancer, it is a good surrogate for judging the effects of androgens on the prostate. In one study of testosterone-treated men, PSA rose to normal levels but no higher than in the controls, leading the authors to conclude that testosterone-induced prostate growth should not preclude hypogonadal men from testosterone replacement therapy.[41] Indeed, another study indicates that even men who achieved supraphysiologic levels of serum testosterone had no significant changes in PSA levels.[42]

In a different evaluation, hypogonadal men with normal pretreatment DRE and serum PSA levels who were treated with parenteral testosterone replacement showed no abnormal alterations in PSA or PSA velocity.[43] The authors concluded that any significant increases in these parameters should not be attributed to testosterone replacement therapy and should be evaluated. The effects of transdermal testosterone replacement on prostate size and PSA levels in hypogonadal men have also been evaluated.[44] Prostate size during therapy with transdermal testosterone was comparable to that reported in normal men, and PSA levels were within the normal range.

### PROSTATE CANCER

There appears to be little association between testosterone replacement therapy and the development of prostate cancer. The etiology of prostate cancer is apparently multifactorial, and dietary, geographic, genetic, and other influences are all thought to play a role in the development of the disease. Recent studies indicate that testosterone levels have no apparent systematic relationship to the incidence of prostate cancer.[45,46]

### SUMMARY

Testosterone is the primary androgenic hormone and is responsible for normal growth and development of male sex organs and maintenance of secondary sex characteristics. Pre-pubertal hypogonadism is generally characterized by infantile genitalia and lack of virilization, while the development of hypogonadism after puberty frequently results in complaints such as diminished libido, erectile dysfunction, infertility, gynecomastia, impaired masculinization, changes in body composition, reductions in body and facial hair, and osteoporosis. Hypogonadal men also report levels of anger, confusion, depression, and fatigue that are significantly higher than those reported in eugonadal men.

Evaluation of potential candidates for testosterone replacement therapy should include a complete medical history and hormonal screening. Total serum testosterone should be measured in the morning. When the serum testosterone level is low and LH is elevated, testosterone replacement therapy is warranted. Patients with low serum LH and testosterone levels need an imaging study of their pituitary and may need endocrinologic consultation.

Testosterone replacement should in theory approximate natural, endogenous production of the hormone. The clinical rationale for treatment of testosterone deficiency may include: stabilizing or increasing bone density; enhancing body composition by increasing muscle strength and reducing adipose tissue; improving energy and mood; and maintaining or restoring secondary sexual characteristics, libido, and erectile function.

Several different types of testosterone replacement are currently marketed, including tablets, injectables, and transdermal systems. Oral testosterone is associated with elevations in liver function tests and abnormalities at liver scan and biopsy. While injectable testosterone is considered safe, fluctuations in testosterone levels may yield variations in libido, sexual function, energy, and mood, and patients may be inconvenienced by the need for frequent testosterone injections. Transdermal systems offer a convenient, though more costly, means of testosterone replacement and have demonstrated safety and efficacy in a number of clinical trials.

The physician prescribing testosterone replacement should evaluate any changes in the clinical symptoms and signs of testosterone deficiency and should assess the patient by performing a DRE and checking serum testosterone levels, PSA, and hematocrit at baseline and at prescribed intervals during treatment. Although testosterone replacement is contraindicated in men with carcinoma of the breast or known or suspected carcinoma of the prostate, in general, therapy appears to be safe for the vast majority of hypogonadal men. There is no apparent association between testosterone replacement therapy and the development of prostate cancer. The administration of exogenous testosterone is not a means of reversing the aging process in men with normal testosterone levels, but it may offer considerable benefit for men suffering from hypogonadism.

### REFERENCES

1. Ghusn, H.F. and Cunningham, G.R.: Evaluation and treatment of androgen deficiency in males. The Endocrinologist, 1(6):399, 1991.
2. American Association of Clinical Endocrinologists and the American College of Endocrinology. AACE Clinical Practice Guidelines for the Evaluation and Treatment of Hypogonadism in Adult Male Patients. ED8143, 1996.
3. Burris, A.S., Banks, S.M., Carter, C.S., Davidson, J.M. and Sherins, R.J.: A long-term, prospective study of the physiologic and behavioral effects of hormone replacement in untreated hypogonadal men. J Androl, 13(4):297, 1992.
4. Seidell, J.C., Bjorntorp, P., Sjostrom, L., Kvist, H. and Sannerstedt, R.: Visceral fat accumulation in men is positively associated with insulin, glucose, and C-peptide levels, but negatively with testosterone levels. Metabolism, 39:897, 1990.
5. Katznelson, L., Rosenthal, D.I., Rosol, M.S., Anderson, E.J., Hayden, D.L., Shoenfeld, D.A. and Klibanski, A.: Using quantitative CT to assess adipose distribution in adult men with acquired hypogonadism. Am J Roentgenol, 170(2):423, 1998.
6. Finkelstein, J.S., Klibanski, A., Neer, R.M., et al.: Osteoporosis in men with idiopathic hypogonadotropic hypogonadism. Ann Intern Med, 106:354, 1987.

7. Stanley, H.L., Schmitt, B.P., Poses, R.M. and Diess, W.P.: Does hypogonadism contribute to the occurrence of a minimal trauma hip fracture in elderly men? J Am Geriatr Soc, 39:766, 1991.
8. Bremner, W.J., Vitiello, M.V. and Prinz, P.N.: Loss of circadian rhythmicity in blood testosterone levels with aging in normal men. J Clin Endocrinol Metab, 56:1278, 1983.
9. Granata, A.R.M., Rochira, V., Lerchl, A., Marrama, P. and Carani, C.: Relationship between sleep-related erections and testosterone levels in men. J Androl, 18:522, 1997.
10. Westaby, D., Ogle, S.J., Paradinas, F.J., et al.: Liver damage from long-term methyltestosterone. Lancet, August 6:261, 1977.
11. McClure, R.D., Oses, R. and Ernest, M.L.: Hypogonadal impotence treated by transdermal testosterone. Urology, 37(3):224, 1991.
12. Snyder, P.J. and Lawrence, D.A.: Treatment of male hypogonadism with testosterone enanthate. J Clin Endocrinol Metab, 51:1335, 1980.
13. Cofrancesco, J. and Dobs, A.S.: Transdermal testosterone delivery systems. The Endocrinologist, 6:207, 1996.
14. Yu, Z., Gupta, S.K., Hwang, S.S., Kipnes, M.S., Mooradian, A.D., Snyder, P.J. and Atkinson, L.E.: Testosterone pharmacokinetics after application of an investigational transdermal system in hypogonadal men. J Clin Pharmacol, 37:1139, 1997.
15. Yu, Z., Gupta, S.K., Hwang, S.S., Cook, D.M., Duckett, M.J. and Atkinson, L.E.: Transdermal testosterone administration in hypogonadal men: Comparison of pharmacokinetics at different sites of application and at the first and fifth days of application. J Clin Pharmacol, 37:1129, 1997.
16. Jordan, W.: Allergy and topical irritation associated with transdermal testosterone administration: A comparison of scrotal and nonscrotal transdermal systems. Am J Contact Derm, 8(2):103, 1997.
17. Jordan, W.P. and Atkinson, L.E.: Comparison of the skin irritation potential of two testosterone transdermal systems: An investigational system and a marketed product. Clin Ther, 20(1):80, 1998.
18. Hajjar, R.R., Kaiser, F.E. and Morley, J.E.: Outcomes of long-term testosterone replacement in older hypogonadal males: a retrospective analysis. J Clin Endocrinol Metab, 82:3793, 1997.
19. Shabsigh, R.: The effects of testosterone on the cavernous tissue and erectile function. World J Urol, 15:21, 1997.
20. Meikle, A.W., Arver, S., Dobs, A.S., Sanders, S.W. and Mazer, N.A.: Androderm: A permeation-enhanced, non-scrotal testosterone transdermal system for the treatment of male hypogonadism. In: Pharmacology, Biology, and Clinical Applications of Androgens. Edited by S. Bhasin. New York, NY: Wiley Liss, Inc., pp. 449-457, 1996.
21. Wang, C.W., Alexander, G., Berman, N., Salehian, B., Davidson, T., McDonald, V., Steiner, B., Hill, L., Callegari, C. and Swerdloff, R.S.: Testosterone replacement therapy improves mood in hypogonadal menóa clinical research center study. J Clin Endocrinol Metab, 81:3578, 1996.
22. Seidman, S.N. and Rabkin, J.G.: Testosterone replacement therapy for hypogonadal men with SSRI-refractory depression. J Affect Disord, 48:157, 1998.
23. Alexander, G.M., Swerdloff, R.S., Wang, C., Davidson, T., McDonald, V., Steiner, B. and Hines, M.: Androgen-behavior correlations in hypogonadal men II. Cognitive abilities. Horm Behav, 33:85, 1998.
24. Tenover, J.S.: Effects of testosterone supplementation in the aging male. J Clin Endocrinol Metab, 75:1092, 1992.
25. Wang, C., Eyre, D.R., Clark, R., Kleinberg, D., Newman, C., Iranmanesh, A., Veldhuis, J., Dudley, R.E., Berman, N., Davidson, T., Barstow, T.J., Sinow, R., Alexander, G. and Swerdloff, R.S.: Sublingual testosterone replacement improves muscle mass and strength, decreases bone resorption, increases bone formation markers in hypogonadal menóa clinical research center study. J Clin Endocrinol Metab, 81:3654, 1996.
26. Bhasin, S., Storer, T.W., Berman, N., Yarasheski, K.E., Clevenger, B., Phillips, J., Lee, W.P., Bunnell, T.J. and Casaburi, R.: Testosterone replacement increases fat-free mass and muscle size in hypogonadal men. J Clin Endocrinol Metab, 82:407, 1997.
27. Sih, R., Morley, J.E., Kaiser, F.E., Perry, H.M., Patrick, P. and Ross, C.: Testosterone replacement in older hypogonadal men: a 12-month randomized controlled trial. J Clin Endocrinol Metab, 82:1661, 1997.
28. Urban, R.J., Bodenburg, Y.H. and Gilkison, C.: Testosterone administration to elderly men increases skeletal muscle strength and protein synthesis. Am J Physiol, 269 (Endocrinol Metab 32): E828ñE826, 1995.
29. Bhasin, S., Storer, T.W., Asbel-Sethi, N., Hays, R., Sinha-Hikim, I., Shen, R., Arver, S. and Beall, G.: Effects of testosterone replacement with a nongenital, transdermal system, Androderm, in human immunodeficiency virus-infected men with low testosterone levels. J Clin Endocrinol Metab, 83:3155, 1998.
30. Miller, K., Corcoran, C., Armstrong, C., Caramelli, K., Anderson, E., Cotton, D., Basgoz, N., Hirschhorn, L., Tuomala, R., Schoenfeld, Daugherty, C., Mazer, N. and Grinspoon, S.: Transdermal testosterone administration in women with acquired immunodeficiency syndrome wasting: a pilot study. J Clin Endocrinol Metab, 83:2717, 1998.
31. Finkelstein, J.S., Klibanski, A., Neer, R.M., Dopplet, S.H., Rosenthal, D.I., Segre, G.V. and Crowley, W.F.: Increases in bone density during treatment of men with idiopathic hypogonadotropic hypogonadism. J Clin Endocrinol Metab, 69:776, 1989.
32. Behre, H.M., Kliesch, S., Leifke, E., Link, T.M. and Nieschlag, E.: Long-term effect of testosterone therapy on bone mineral density in hypogonadal men. J Clin Endocrinol Metab, 82:2386, 1997.
33. Leifke, E., Korner, H.C., Link, T.M., Behre, H.M., Peters, P.E. and Nieschlag, E.: Effects of testosterone replacement on cortical and trabecular bone mineral density, vertebral body area and paraspinal muscle area in hypogonadal men. Eur J Endocrinol, 138:51, 1998.

34. Burstein, S., Grumbach, M.M. and Kaplan, S.L.: Early determination of androgen-responsiveness is important in the management of microphallus. Lancet, 2:983, 1979.
35. Taur, H., Shafir, R., Schachar, J., et al.: Microphallic hypospadius: testosterone therapy prior to surgical repair. Br J Plast Surg, 36:398, 1983.
36. World Health Organization. Contraceptive efficacy of testosterone-induced azoospermia in normal men. World Health Organization Task Force on methods for the regulation of male fertility. Lancet, 336:955, 1990.
37. World Health Organization. Contraceptive efficacy of testosterone-induced azoospermia and oligozoopermia in normal men. Fertil Steril, 65:821, 1996.
38. Tan, K.C., Shiu, S.W., Pang, R.W. and Kung, A.W.: Effects of testosterone replacement on HDL subfractions and apolipoprotein A-I containing lipoproteins. Clin Endocrinol (Oxf), 48:187, 1998.
39. Anderson, F.H., Francis, R.M. and Faulkner, K.: Androgen supplementation in eugonadal men with osteoporosis(effects of 6 months of treatment on bone mineral density and cardiovascular risk factors). Bone, 18:171, 1996.
40. Arver, S., Meikle, A.W., Dobs, A.S., et al.: Hypogonadal men treated with the Androderm testosterone transdermal system had fewer abnormal hematocrit evaluations than those treated with testosterone enanthate injections. In: Program and Abstracts of the 77th Annual Meeting of the Endocrine Society, Washington DC, June 14-17, 1995, Abstract P1-327.
41. Behre, H.M., Bohmeyer, J. and Nieschlag, E.: Prostate volume in testosterone-treated and untreated hypogonadal men in comparison to age-matched, normal controls. Clin Endocrinol (Oxf), 40:341, 1994.
42. Cooper, C.S., MacIndoe, J.H., Perry, P.J., Yates, W.R. and Williams, R.D.: The effect of exogenous testosterone on total and free prostate specific antigen levels in healthy young men. J Urol, 156:438, 1996.
43. Svetec, D.A., Canby, E.D., Thompson, I.M. and Sabanegh, E.S.: The effect of parenteral testosterone replacement on prostate specific antigen in hypogonadal men with erectile dysfunction. J Urol, 158:1775, 1997.
44. Meikle, A.W., Arver, S., Dobs, A.S., Adolfsson, J., Sanders, S.W., Middleton, R.G., Stephenson, R.A., Hoover, D.R., Rajaram, L. and Mazer, NA. Prostate size in hypogonadal men treated with a nonscotal permeation-enhanced testosterone transdermal system. Urology 1997; 49:191-6.
45. Ebling DW, Ruffer J, Whittington R, Vanarsdalen K, Broderick GA, Malkowicz SB, Wein AJ. Development of prostate cancer after pituitary dysfunction: A report of 8 patients. Urology 1997;49:564-568.
46. Gustafsson O, Norming U, Gustafsson S, Eneroth P, Astrom G, Nyman CR. Dihydrotestosterone and testosterone levels in men screened for prostate cancer: a study of a randomized population. Br J Urol 1996;77:433-440.

**This article should be referenced as follows:**

Hellstrom, W.J.G. (2004) Testosterone replacement therapy. *TheScientificWorldJOURNAL* **4 (S1)**, 142–149.

**Handling Editor:**

Anthony Atala, Principle Editor for *Urology* — a domain of *TheScientificWorldJOURNAL.*
